# First person – Santu Saha

**DOI:** 10.1242/bio.059987

**Published:** 2023-05-25

## Abstract

First Person is a series of interviews with the first authors of a selection of papers published in Biology Open, helping researchers promote themselves alongside their papers. Santu Saha is first author on ‘ Bioengineering of a tumour-stroma 3D-tumouroid co-culture model of hypopharyngeal cancer’, published in BiO. Santu conducted the research described in this article while a Newton International Fellow of Academy of Medical Sciences (Research Associate) in Professor Nicola Curtin's lab at Newcastle University. He is now a Research Associate in the lab of Professor Christine Harrison at Leukaemia Research Cytogenetics Group at Newcastle University. He is a passionate early-career translational cancer researcher and his long-term research interest is in exploring gene – drug association based targeted cancer therapy through exploiting novel tools.


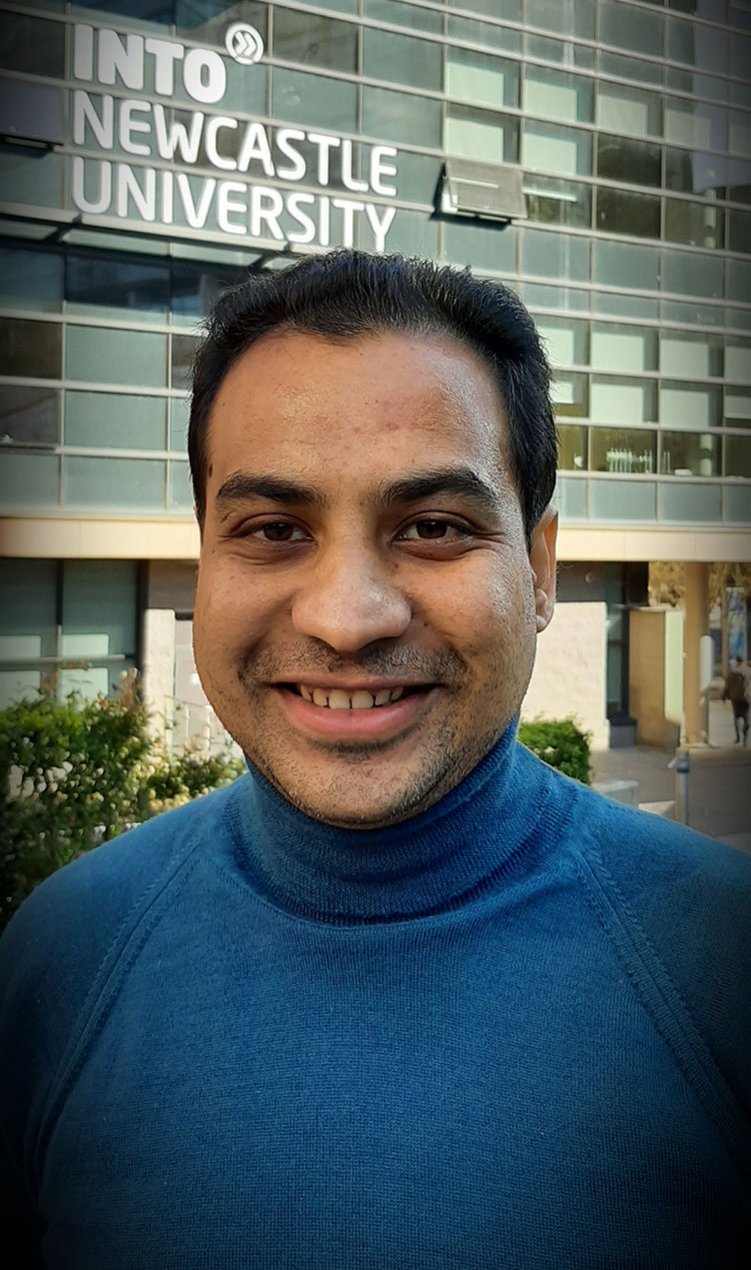


**Santu Saha**

**Describe your scientific journey and your current research focus**

In 2014, I finished my PhD from Kalyani University, India, and after working with cancer cell lines to investigate the anticancer potential of plant natural products; I became more interested in studying cancer biomarker(s), their regulatory mechanisms and therapeutic interventions. To complement my research interest, I obtained a postdoctoral fellowship funded by the Department of Biotechnology (DBT) of the Government of India to work in head and neck cancer at Chittaranjan National Cancer Institute (CNCI), India (2015-2017). Unfortunately, due to lack of any follow-on funding, I had to stop my research and I joined the Tata Medical Center, India, as a Bio-bank Technologist (2017-2018). During that tenure, I made my first visit to Newcastle University through a travel grant funded by the Union for International Cancer Control (UICC) and that visit motivated me to work in translational cancer research. Yet, the extended period of away from my research work continued until 2019 and during that phase, I was passionately applying for my own international research fellowships. However, that was not easy and after five unsuccessful applications (e.g. two attempts at H2020 Marie Sklodowska-Curie Actions, the Humboldt Research Fellowship, the Open Topic Postdoc Position of TU Dresden Germany, and the Committee on Clinical Pharmacology and Pharmacogenomics program of University of Chicago, IL, USA), on my sixth attempt I obtained the Newton International Fellowship. That was the turning point of my life and I decided to move in the UK to re-start my research from 2019, which I am continuing now. Within my post-PhD 6 years of active research activity so far, I am honored to have received the some grants and awards from prestigious bodies including the Wellcome Trust, the European Society for Radiotherapy and Oncology (ESTRO), H2020 Marie Sklodowska-Curie Actions Seal of Excellence that keep motivating me to go through the roller coaster ride in the academic research. Recently, I have started a new project in Acute Lymphoblastic Leukaemia (ALL) at the Leukaemia Research Cytogenetics Group, Newcastle University. The aim of this project is to establish the analysis pipelines for the drug-gene association matrix. My project requires extensive understanding in anticancer drug screening and bioinformatics. From my previous projects (Newton International Fellowship and Wellcome Trust Translational Partnership grants), I have gained knowledge in anticancer drug toxicity studies in 2D/3D cell culture/co-culture and mice models. Here, I want to develop my expertise in bioinformatics through obtaining proper training in computer programming languages (e.g. R and Python).

**Who or what inspired you to become a scientist?**

During my high schooling days, I wanted to study medicine to fulfill my long-term goal of contributing to the health and wellbeing of people. However, being unsuccessful to pursue that through the competitive exam route, my direction shifted to become a scientist to fulfill my long-term goal.

**How would you explain the main finding of your paper?**

Head and neck cancer (HNC) differs at anatomical sites and hypopharyngeal cancer (HPC) is a type of HNC. The non-surgical treatment option for advanced cases of HPC is radiotherapy (RT) with/without chemotherapy but survival is poor (25-30%). Thus, more laboratory-based research is essential to discover new treatment approaches in combination with RT. Yet, obtaining post-RT treated tumour specimens and lack of animal models with identical anatomical sites are the major research barriers. A tumour is a complex structure of many components that is called the tumour microenvironment (TME) and RT induces complex response when striking the TME to either boost or suppress the immunity. Altered immunity has an effect on variable RT response and to achieve a more durable anti-tumour immune response, a new type of immune-modulatory drug, called the immunotherapy, would be beneficial. Thus, studying the effect of RT±anti-cancer drugs by culturing only cancer cells on flat surfaces is not always a good approach to discover new combination of treatment with RT. To circumvent these obstacles, we have developed a novel tool by growing HPC cells with immortal non-cancer cells of bone marrow origin to help aggregating the HPC cells together forming a 3D tumour-like structure in a Petri-dish and named this tool “3D-tumouroid co-culture”. The wider application of this pre-clinical research tool is in understanding newer combination (e.g. immunotherapy) treatment approaches with RT in HPC and beyond.

“…we have developed a novel tool by growing HPC cells with immortal non-cancer cells of bone marrow origin to help aggregating the HPC cells together forming a 3D tumour-like structure in a Petri-dish…”

**What are the potential implications of this finding for your field of research?**

Unlike the conventional platinum-based chemo-radiotherapy (CRT) treatment from the standard guidelines of CRT in HNC or HPC which shows variable response, our innovative research tool will allow rationale-based testing of new classes of drugs and immunotherapy as a novel combinations with the most advanced RT approaches (e.g. proton/carbon beam therapy). These novel therapy combination could provide treatment benefit to those patients who cannot receive platinum drugs with RT due to renal inefficiency and or have unresectable tumours receiving only RT with potentially poor survival.

**Figure:**
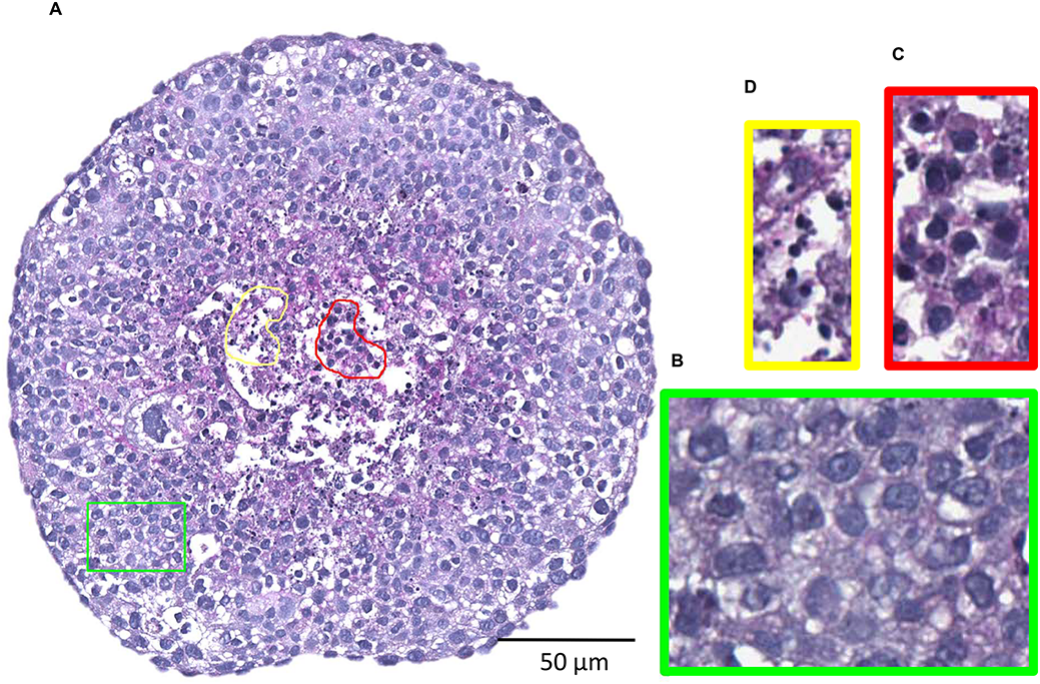
***Histological characterisation of a 3D-tumouroid:* (A) Cancer cells FaDu and bone stromal cells HS-5 were grown together in a special type of very small welled culture plate (a type of petri-dish) for 10 days to form a 3D-tumour like structure called the tumouroid.** Later on, the tumouroid was fixed in paraffin (a type of wax) and then sectioned with a very fine blade using an instrument called microtome. PAS stained section of the tumouroid was scanned using a sophisticated image scanner. From the whole image of the tumouroid (10X objective), regions were annotated and presented separately in coloured boxes (40X objective) to clearly distinguish different cell types. (B-D) Cancer cells FaDu (green coloured box), bone stromal cells HS-5 (red coloured box) and necrotic (yellow coloured box) cells.

**Which part of this research project was the most rewarding?**

This project was built on my Newton International Fellowship (NIF) and I conceptualised this project to apply for any external fellowships/grant applications to go beyond my NIF during the time of Covid-19 pandemic, when our university was completely closed. Defending many internal criticisms, I started working on this project gradually with my co-author Rachel Howarth after our lab-reopened partially in mid-June 2020. However, it was difficult to complete any long experiments at that time due to limited working times of 5 hour shifts each day with only 20% capacity in the “working in a bubble” pattern, restrictions to out of hours work, and limited or no access to some instruments. Despite these obstacles, after producing some preliminary data I realised a clinical collaboration would be essential for the long-term translational impact of this project. At that point, our co-author Charles Kelly showed his interest and we started applying for external funding to support my salary to cover my no-cost extension period of NIF that ended in January 2022. The project was at its utmost challenging period when I reduced my working hours due to parental responsibilities to our first boy born in September 2020 followed by a couple of months later when I was going through an extremely emotional phase due to loss of my father thousands of miles away from the UK in Kolkata, India. It was not until September-October 2021, when the project received some oxygen after receiving funding through the Wellcome Trust Translational Partnership award (WTTP) to cover my salary at 40% full-time equivalent (FTE) and other 40% FTE came through an internal funding to our co-author Sweta Sharma-Saha. Unfortunately, even after several attempts to different funders no further follow-on funding was received and at the end of my contract in May 2022, I decided to compile the work for publication rather waiting for further funding. The project on its first attempt for publication received some important reviews from a journal that belongs to a more than 100-year-old, prestigious research body in the UK. Those reviews gave me further confidence in our research work and I decided to address those reviews to enrich the quality of the paper and then to submit via the fast-track review process (which I found very unique in its quality) to Biology Open. After the acceptance of our work for publication and now as I answer the interview questions, I am feel all those things are worth mentioning as most rewarding experiences.

**What do you enjoy most about being an early-career researcher?**

I keep learning from my failures at this early-career stage of my research.

**What piece of advice would you give to the next generation of researchers?**

I am too early in the process of learning to advise anything to the next generation of researchers. However, I can share my personal experience to be that resilience is something important in academic research.

**What's next for you?**

Apart from my recent research activities mentioned above, I am also upgrading my teaching skills and for that, I have started preparation to apply for the UK Professional Standards Framework's (UKPSF) Associate Fellowship through the Experiential Route.
